# SARS-CoV-2 Spike Protein Interaction Space

**DOI:** 10.3390/ijms241512058

**Published:** 2023-07-27

**Authors:** Claudiu N. Lungu, Mihai V. Putz

**Affiliations:** 1 Department of Morphological and Functional Science, University of Medicine and Pharmacy Dunarea de Jos, Str. Alexandru Ioan Cuza No. 36, 800017 Galati, Romania; lunguclaudiu5555@gmail.com; 2 Laboratory of Structural and Computational Physical-Chemistry for Nanosciences and QSAR, Biology-Chemistry Department, Faculty of Chemistry, Biology, Geography, West University of Timisoara, Str. Pestalozzi No. 16, 300115 Timisoara, Romania

**Keywords:** COVID-19, antibody, paratope, QSAR, chemical space, spike protein, SARS-CoV-2, antibody binding

## Abstract

Severe acute respiratory syndrome coronavirus 2 (SARS-CoV-2) is a +sense single-strand RNA virus. The virus has four major surface proteins: spike (S), envelope (E), membrane (M), and nucleocapsid (N), respectively. The constitutive proteins present a high grade of symmetry. Identifying a binding site is difficult. The virion is approximately 50–200 nm in diameter. Angiotensin-converting enzyme 2 (ACE2) acts as the cell receptor for the virus. SARS-CoV-2 has an increased affinity to human ACE2 compared with the original SAR strain. Topological space, and its symmetry, is a critical component in molecular interactions. By exploring this space, a suitable ligand space can be characterized accordingly. A spike protein (S) computational model in a complex with ACE 2 was generated using silica methods. Topological spaces were probed using high computational throughput screening techniques to identify and characterize the topological space of both SARS and SARS-CoV-2 spike protein and its ligand space. In order to identify the symmetry clusters, computational analysis techniques, together with statistical analysis, were utilized. The computations are based on crystallographic protein data bank PDB-based models of constitutive proteins. Cartesian coordinates of component atoms and some cluster maps were generated and analyzed. Dihedral angles were used in order to compute a topological receptor space. This computational study uses a multimodal representation of spike protein interactions with some fragment proteins. The chemical space of the receptors (a dimensional volume) suggests the relevance of the receptor as a drug target. The spike protein S of SARS and SARS-CoV-2 is analyzed and compared. The results suggest a mirror symmetry of SARS and SARS-CoV-2 spike proteins. The results show thatSARS-CoV-2 space is variable and has a distinct topology. In conclusion, surface proteins grant virion variability and symmetry in interactions with a potential complementary target (protein, antibody, ligand). The mirror symmetry of dihedral angle clusters determines a high specificity of the receptor space.

## 1. Introduction

Designing an antibody against the SARS-CoV-2 spike protein S is challenging due to its nonspecific nature, mainly conferred by each spike protein monomer [1]. The spike protein (S protein) is the most solvent from all virus surface proteins. In addition, the S protein is a trimer [2,3]. The inverse tetrahedron’s unique geometry confers a maximum solvent exposure surface. In addition, the mobile domains of the pyramid base confer the COVID-19 surface protein structural mobility. Furthermore, mobile structural parts are subject to variable mutations [4].

Recent research and protein sequences designed to fit spike protein S failed to bind. A lack of specificity is a result of high mobility and flexibility combined with additional maximum solvent exposure of the monomer domains [5]. 

A mass effect should virtually resolve this lack of specificity; in other words, by increasing the mass of the desired paratope, that will interact with the epitope (S protein), an alternative unavailable from the receptor–ligand interaction point of view [6]. 

Judging from the antigen–antibody point of view, epitope amino acid (Aa) sequences located on the antigen must fit complementarily with the Aa chain (usually between 5 and 10) of the paratope (antigen variable (VL) and constant regions (CR)) [7].

In mathematics, polynomial expressions are based on indeterminants and coefficients. Polynomials involve operations like addition, subtraction, and multiplication. Polynomials are involved in many science areas. For example, in chemistry, they are used to set ranges. There are two types of polynomials: real and complex polynomials, respectively. The real polynomial has real roots, while complex ones have complex roots [8]. A polynomial function is an operation that can be characterized by computing a polynomial. A function (*f*) of one argument from a given domain is a polynomial function if a polynomial exists. Like any other functions, polynomial functions can be represented by a graph [9]. The discriminant of a polynomial is a quantity characterized by the coefficient and allows the deduction of some properties of the roots (i.e., it is a polynomial function of the coefficient of the original polynomial) [10]. The regular use of discriminants in algebraic geometry is for characterizing plane algebraic curves and algebraic hypersurfaces. If V is such a curve or hypersurface, V is characterized as the zero-set polynomial. This polynomial is regarded as a univariate polynomial in one indeterminate, with polynomials in the other indeterminates as coefficients. The discriminant concerning the selected indeterminate shows a hypersurface W in the space of the others. The points of W are the projection of the points of V, which are singular or have a tangent hyperplane parallel to the axis of the selected indeterminate, respectively [11].

Suppose *f* is a bivariate polynomial with real coefficients. If *f*  = 0 is the implicit equation of a real plane algebraic curve, the discriminant is a polynomial where roots are the x-coordinates of the singular points. In other words, the computation of the roots allows the computation of all of the remarkable points of the curve [12].

The logarithm is a reverse function of exponentiation, meaning that the logarithm of a particular number x is the exponent to which another fixed number must be raised to produce that number x. John Napier introduced logarithms in 1614 as a means of simplifying calculations. Navigators rapidly adopted them to perform high-accuracy computations more efficiently [13]. Logarithmic scales reduce broad quantities to smaller ones. In chemistry, pH is a logarithmic measure for the acidity of an aqueous solution. Logarithms are commonplace in scientific formulae and measurements of the complexity of algorithms and geometric objects called fractals. From the perspective of group theory, the identity log(cd) = log(c) + log(d) shows a group isomorphism among positive reals under multiplication and reals under addition [14]. Logarithmic functions are the single continuous isomorphisms between these groups. Using that isomorphism, the Haar (Lebesgue) dx on the reals corresponds to the Haar measure dx/x on the positive reals. The non-negative reals not only have a multiplication but also have an addition, and they form a semiring called the probability semiring; this is a semifield. The concept of the logarithm as the inverse of exponentiation also extends to other mathematical structures. However, the logarithm tends to be a multi-valued function in general settings.

The logarithm is associated with the natural logarithm: Lis (z) = −ln(1 − z). Moreover, Lisequals the Riemann Zeta function ζ(s) [15].

The domain of a function is the array of inputs allowed by the function. Furthermore, the domain of a function is the set of numbers that can go into a given function. The set of possible y-values is called the range [16].

Regarding a spatial perspective for a linked oriented manifold M of dimension 2n, the intersection form is defined on the n-th cohomology group by evaluating the cup product on the entire class [M] in H2n(M, ∂M). Stated precisely, this is a bilinear form. This is a symmetric form for n even (so, 2n = 4k is doubly even), in which case the signature of M is defined to be the form’s signature, and an alternating form for n odd (so, 2n = 4k + 2 is singly even). These can be referred to uniformly as ε-symmetric forms, where ε = (−1)n = ±1, respectively, for symmetric and skew-symmetric forms [17]. These forms are essential topological invariants. If possible, choose representative n-dimensional submanifolds A and B for the Poincaré duals of a and b. Then, λM (a, b) is the oriented intersection number of A and B, which is well defined because the dimensions of A and B sum to the total dimension of M; they generically intersect at isolated points. This explains the terminology intersection form. In algebraic geometry, the Chow groups (named after Wei-Liang Chow) by Claude Chevalley (1958) of an algebraic variety over any field are algebro-geometric analogs of the homology of a topological space. The elements of the Chow group are formed out of subvarieties (so-called algebraic cycles), similarly to how simplicial or cellular homology groups are formed out of subcomplexes. When the variety is smooth, the Chow groups can be interpreted as cohomology groups (compared with Poincaré duality) and have a multiplication called the intersection product [18]. The Chow groups carry rich information about an algebraic variety and are generally hard to compute. In mathematics, specifically in algebraic geometry, the Grothendieck–Riemann–Roch theorem is an example of coherent cohomology [19]. It is a generalization of the Hirzebruch–Riemann–Roch theorem about complex manifolds, which is itselfa generalization of the classical Riemann–Roch theorem for line bundles on compact Riemann surfaces [20]. Riemann–Roch-type theorems relate Euler characteristics of the cohomology of a vector bundle with their topological degrees or, more generally, their characteristic classes in (co)homology or algebraic analogs. The classical Riemann–Roch theorem does this for curves and line bundles, whereas the Hirzebruch–Riemann–Roch theorem generalizes this to vector bundles over manifolds. The Grothendieck–Riemann–Roch theorem sets both theorems in a relative situation of a morphism between two manifolds (or more general schemes) [21]. 

A molecule’s three-dimensional arrangement influences molecular properties like reactivity and biological activity [22]. The geometry of a molecule is specified by employing bond lengths, bond angles, and dihedral angles, respectively [23]. Bond length is the average distance between two atoms’ nuclei that are bound together. The bond angle is the angle formed by three atoms involving two bounds. A mathematical relationship between the bond angles for a central atom and four peripheral atoms is expressed using a determinant. The cos of the bond angles of each atom involved is used to build the determinant’s matrix. If *θ* is the determinant, and cos*θ* represents the bound angle value in Å, and if cos*θ*11, cos*θ*22, cos*θ*33, and cos*θ*44 are considered zero, the determinant matrix can be written as follows [24]:

$$\theta =\begin{array}{cccc} \cos\theta 11 & \cos\theta 12 & \cos\theta 13 & \cos\theta 14\\ \cos\theta 21 & \cos\theta 22 & \cos\theta 23 & \cos\theta 24\\ \cos\theta 31 & \cos\theta 32 & \cos\theta 33 & \cos\theta 34\\ \cos\theta 41 & \cos\theta 42 & \cos\theta 43 & \cos\theta 44 \end{array}$$

The dihedral angle is formed by intersecting two planes. Notably, in chemistry, it is the angle formed by two sets of atoms, i.e., two planes with two common atoms. Torsion angles are a particular example of dihedral angles in chemistry. Geometric relationships of particular atoms united by a chemical bound are described. Three non-colinear atoms that form an angle are considered to be in the same plane [25].

Regarding chemical compounds, by intersecting two distinct planes, a dihedral angle is formed [26]. Molecular conformations are characterized using dihedral angles. Specifically, stereochemical arrangements are used. Arrangements equivalent to angles between 0° and ±90° are called syn (s), and those equivalent to angles between ±90° and 180° are called anti (a). Similarly, arrangements equivalent to angles between 30° and 150° or between −30° and −150° are called clinal (c), and those between 0° and ±30° or ±150° and 180° are called periplanar (p). Four ranges of angles are defined: 0° to ±30° is colled synperiplanar (sp); ranges 30° to 90° and −30° to −90°are called synclinal (sc); ranges 90° to 150° and −90° to −150° are called anticlinal (ac); ±150° to 180°is called antiperiplanar (ap) [27,28]. 

In proteins, three dihedral angles are described for a chain: φ (phi), ψ (psi), and ω (omega). The planarity of the bond usually restricts ω to 180° (the typical trans case) or 0° (the rare cis case). The space between the Cα atoms within the trans and cis isomers is approximately 3.8 and 2.9 Å, respectively. The overwhelming majority of the peptide bonds in proteins are trans [29].

The sidechain dihedral angles are designated with χn (chi-n). They tend to cluster near 180°, 60°, and −60°, which are called the trans, gauche+, and gauche− conformations. The soundness of certain side chain dihedral angles is laid low with the values φ and ψ. For example, there are direct steric interactions between the Cγ of the side chain within the gauche+ rotamer and the backbone nitrogen of the following residue when ψ is near −60° [30,31,32].

The polynomial equation of Ramachandran plot values can be represented as a surface of revolution. A surface of revolution could be a surface in Euclidean space created by rotating a curve (the generatrix) around an axis of rotation. To generate a surface of revolution out of any two-dimensional scalar function y = *f*(x), make u the function’s parameter, set the axis of rotation’s function to easily u, then use v to rotate the function round the axis by setting the opposite two functions up to f(u) sin v and f(u) cos v. As an example, to rotate a function y = *f*(x) around the x-axis ranging from the highest of the xz-plane, parameterize it as [33]:

r(u,vfo) = (u,f(u)sinv, f(u) cos v) for u = x and vЄ [0,2pi]

However, in order to normalize the dihedral angle values to characterize the dihedral angle clusters, alogarithmic trendline may be a best-fit curved line that is most useful when the speed of change within the data increases or decreases quickly and then levels out. A logarithmic trendline can use negative and positive values [34].

Lastly, protein symmetry can be classified by crystallographic point groups. The cyclic groups have one rotational axis symmetry, C1 symmetry (monomeric protein), and C2 symmetry (dimeric protein). These proteins have functions that require directionality, such as channel formation and interactions with membranes [35]. The dihedral group has higher symmetry containing an additional perpendicular axis of two-fold symmetry. This symmetry provides increased allosteric control. The cubic group shows a three-fold symmetry combined with a nonarticular rotational axis. This group is associated with storage and transport proteins (icosahedral symmetry) [36].

Also, some in silico studies propose new molecules that can bind efficiently to SARS-CoV-2. In this respect, curcuminoids had high binding affinity [37]. Another class of compounds of interest is the turmeric-derived compounds that act against RNA-dependentRNA polymerase of SARS-CoV-2. These compounds showed promising perspectives to be designed as RdRp-RNA inhibitors [38]. Also, some natural compounds have been considered SARS-CoV-2 inhibitors, such as compounds derived from Nigella Sativa. Alpha dederin, rutin, and nigellamine A2showed promising binding energy with the specific SARS-CoV-2 proteins [39].

In conclusion, protein 3D structure IDs were explored to gain insights into spike protein chemical space and antigen interaction. The coronavirus main protein is spike protein (S), a trimer. Also, the envelope protein(E), a pentamer composed of five equivalent units, and membrane protein (M),a dimer, are discussed [40]. 

Lastly, some proposed first-,second-, and third-generation vaccines have failed to produce long-lasting antibodies. This computational study illustrates the COVID-19 spike protein S interaction space and proposes some Aa sequences (see also Supplementary Materials File S1) that can be conceivably used as a paratope in designing an effective COVID-19 vaccine.

## 2. Results

Light chains (VL) interactions (structures retrieved from the literature) with an epitope (structures retrieved from the literature) are presented in Table 1. Also, ACE II interactions with the spike protein monomer and with itself is included in the table.

**Figure 1:**
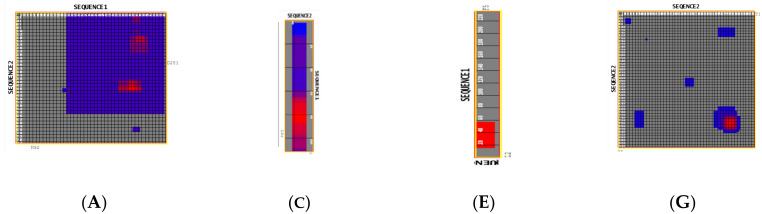

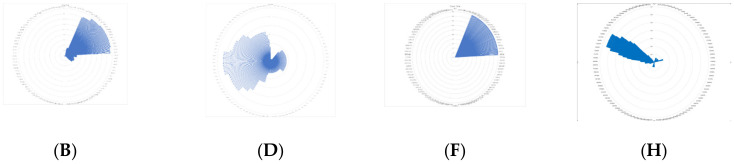
(**A**) I-Frag interaction map between ACEII monomer and spike protein monomer (6VXX) based on I-Frag interaction scores; (**B**) Radar plot based on I-Frag interaction scores between ACE II monomer and 6VXX monomer; (**C**,**D**) 6VXX sequence1A. I-Frag interaction map between 6VXX monomer and Aa based on I-Frag interaction scores; (**D**). Radar plot based on I-Frag interaction scores between 6VXX monomer and sequence1; (**E**) 6CVR sequence1 Frag interaction map between 6CVR monomer and sequence1 based on I frag interaction scores; (**F**). Radar plot based on I-Frag interaction scores between 6CVR monomer and sequence1; (**G**) I-Frag interaction map between 6VXX monomer and 6VXX based on I frag interaction scores; (**H**) Radar plot based on I-Frag interaction scores between 6VXX monomer and 6VXX monomer 6VXX monomer.

Two-dimensional interaction plots and scatter plots of molecular interactions are represented in Figure 1.

**Figure 2:**
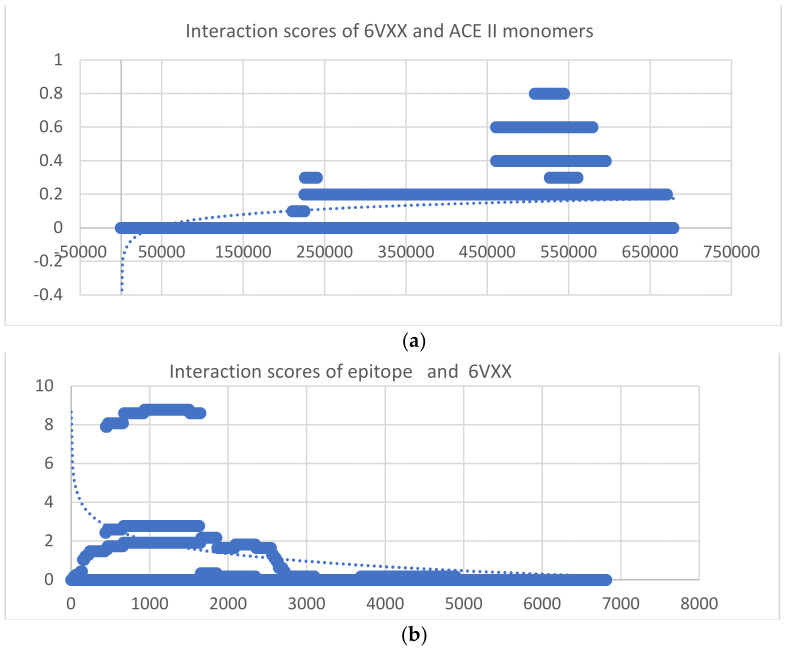

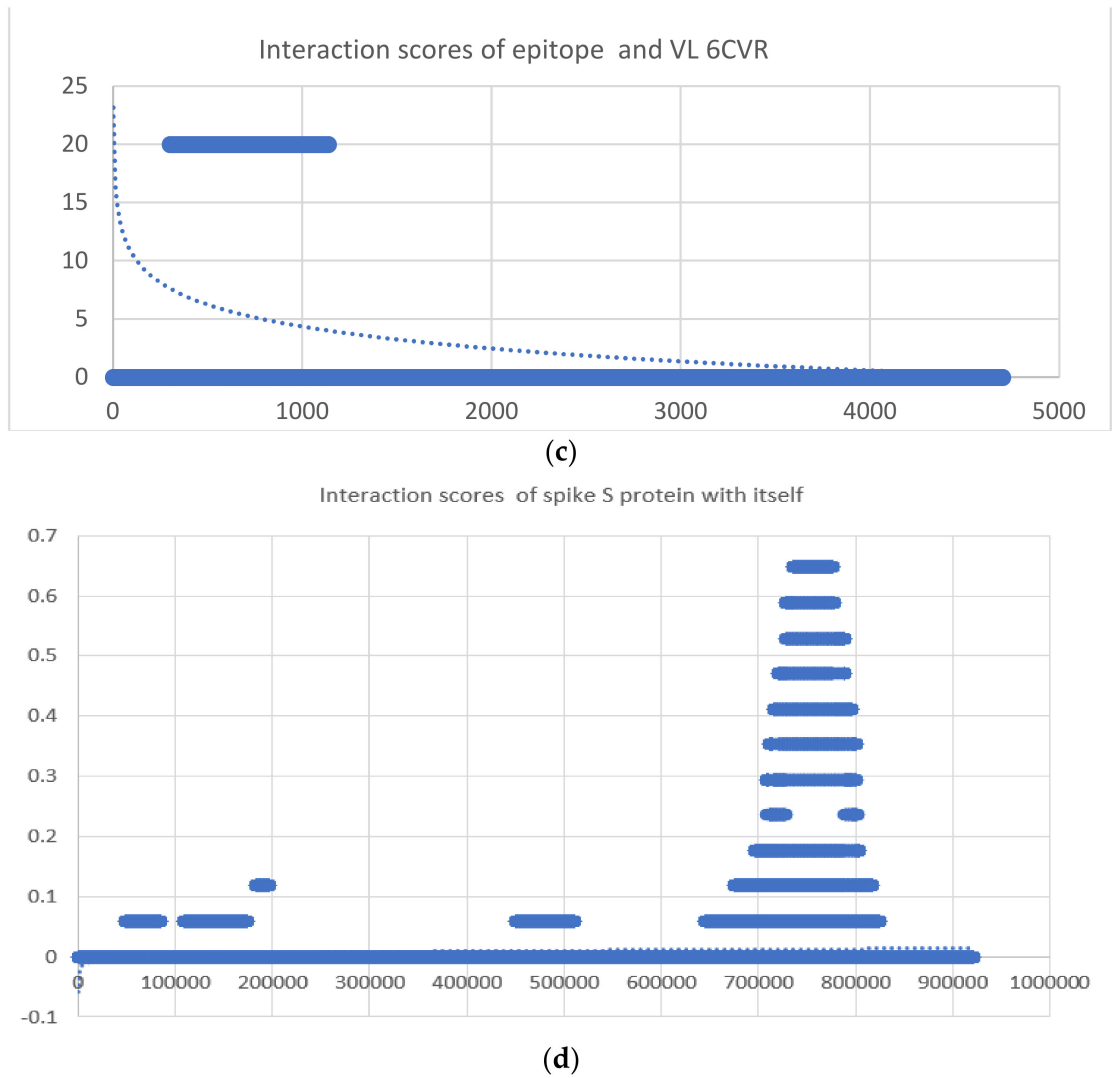
Scatter plots representing: (**a**) I Frag score results from interaction between the ACEII monomer and 6VXX monomer, characterized by 678,370 interaction pairs between one Aa; from 6VXX and one Aa; from ACE II (OX axes). The I Frag score corresponding to each pair of Aa is represented on oy axes. A logarithmic trendline (dash points) is also drawn. (**b**) 6VXX interaction with sequence1 is characterized by almost 7000 Aa pairs interacting (OX axes). The I Frag score corresponding to each pair of Aa is represented on OY axes. A logarithmic trendline (dash points) is also drawn (I Frag scores values significative of stronger interactions). (**c**) Aa sequence (epitope) interaction with 6CVR. (**d**) Spike protein interaction with itself.

In Figure 2, molecular interactions are represented using the I-Frag interaction score.

The Aa sequence (epitope) interaction with 6CVR is outlined by a significant I Frag score (ox axes—up to 20) and fewer Aa interaction pairs (<5000). Also, there are two domains of interaction only: one with I frag scores of 20 and an indifferent domain with no I frag values. The spike protein S sequence interacts with an identical spike protein S sequence. As observed, multiple domains of interactions are observed. As seen in the ACEII–6VXX interaction, a dominant domain with a lower interaction score is observed together with small domains.

Due to similar discriminant values (in interactions with the spike protein monomer), the Aa sequence 6VXX was used as a template. The sequence was searched in the PDB database [41], with the following results retrieved: 6cwt.1.Ea, a capsid protein HBV(100% identity); 6cwd.1.B, a capsid protein HIV (98.40% identity); 6cwd.1.D, a capsid protein HBV (98.40% identity) [42]; 6cbv.1.D, a cacapsidnterovirus protein (identity 98.40) [43]. The sequence with 100% identity is shown below.

ELVMTQTPSSTSAAVGGTVTINCQASQSIGNALAWYQQKPGQPPKLLISAGSNLASGVPS RFRGSGSGTEYTLTISDVQREDAATYYCLGTYSAIDRAFGAGTNVEIERTVIDPYKEFGA TVELLSFLPSDFFPSVRDLLDTAAALYRDALESPEHASPHHTALRQAILCWGDLMTLATW

**Figure 3:**
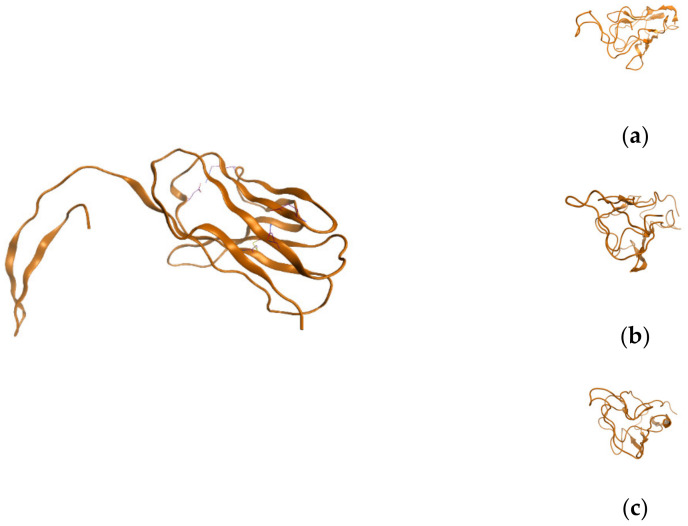
Homology model of the sequence, together with three favorable structural conformations: (**a**–**c**).

The sequence was scanned for paratopes to find binding sites using the Paratome server. The Paratore server retrieved for this sequence the following results (Table 2):

Furthermore, using the discussed sequence, a homology model was performed using the SWISS protserver, and the following homology model was retrieved (Figure 3). Also, the value of potential energy of conformations (E), strain energy (dE), conformation stereochemistry integer (chi), radius of gyration (gry), and globularity of the conformation (glob) was computed as follows: (a) PE = −2077.8716, dE = 0.4685, Chi = 1, Rgir = 14.5243, Glob = 0.4600, ecc = 0.9628; (b) E = −2078.3401, dE = 0.0000, Chi = 1, Rgir = 14.3664, Glob = 0.4691, ecc = 0.9873; (c) E = −2075.5857, dE = 2.75451, Chi = 1, Rgir = 13.8382, Glob = 0.5646, ecc = 0.9513 (Figure 3).

**Figure 4:**
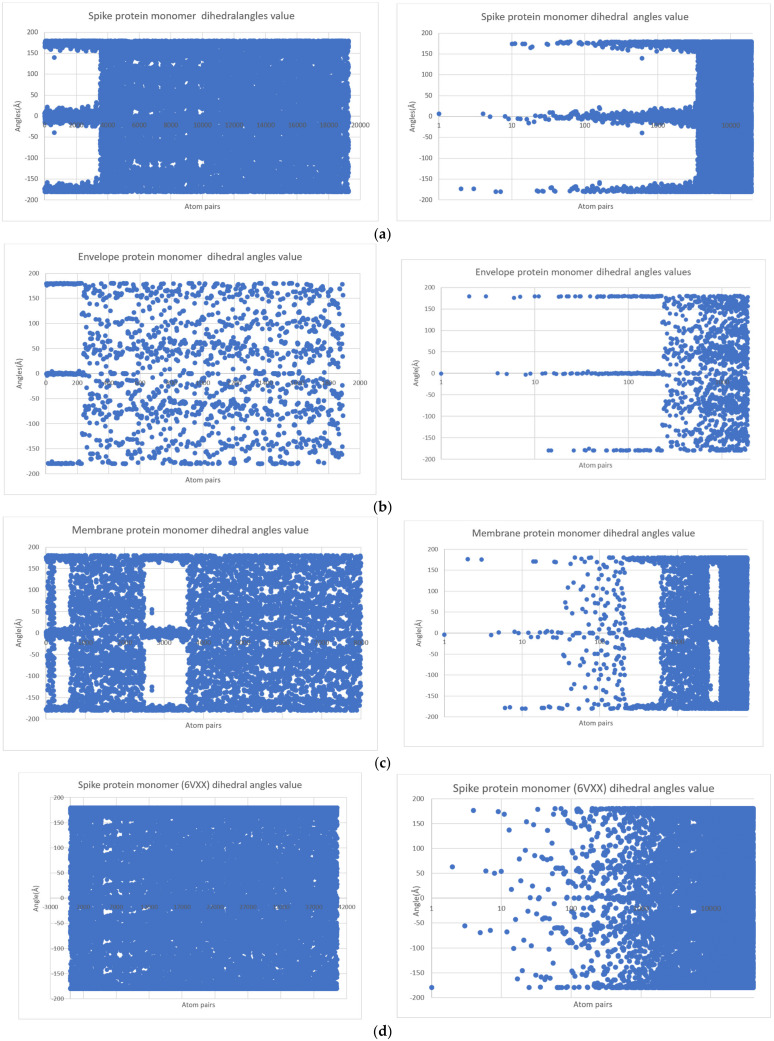
Dihedral angles of surface protein monomers. On the right, dihedral angles are represented after the log representation of the ox axis. (**a**) Spike protein monomer of SARS dihedral angle population; (**b**) envelope protein monomer of dihedral angle population of SARS-CoV-2; (**c**) membrane protein monomer of dihedral angle population of SARS-CoV-2; (**d**) spike protein monomer of dihedral angle population of SARS-CoV-2.

Furthermore, dihedral angles computed in silico for each protein monomer are represented in Figure 4.

As shown in Figure 4a, for the spike protein of SARS, three dihedral angle clusters are observed: two symmetrical clusters in the range 1600–1800 and −1600–(−1800), respectively, and a central domain in the range −300–300. A population-abundant baseline domain is also observed. Envelope protein monomer dihedral angle population shows two symmetrical clusters in the range 1700–1800 and −1700–(−1800), respectively. A central cluster around 00 is also observed (Figure 4b). The membrane protein monomer dihedral angle population shows symmetrical clusters in the range 1600–1800 and −1600–(−1800), respectively. Also, a central cluster in the range 00–100 and −100–00 is described. Two central continuous dihedral angle population domains are observed (Figure 4c). 

In Figure 4d, spike protein monomer of SARS-CoV-2 is represented. In contrast with the dihedral angle populations of protein, the monomers discussed have two symmetrical clusters in the range 1600–1700 and −1600–(−1700), respectively, and another two symmetrical clusters in the range 600–700 and −600–(−700), respectively. Also, a central domain situated around the 00 value is observed. Two baselines are also observed. 

Logarithmic dihedral angles population trendlines equations for each protein monomer are represented below in Table 3.

**Figure 5:**
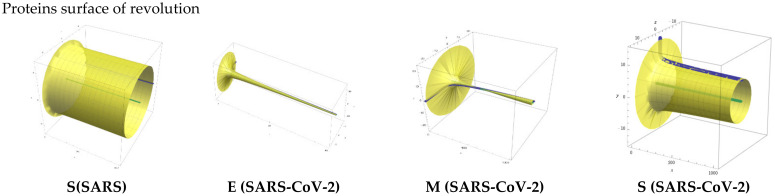
Protein monomers surface of revolution. The surface of resolution is generated using the logarithmic trendline dihedral angles equation. The axis of revolution is represented in green. The generator (dihedral angle trendline equation) is represented in blue (see also Supplementary Materials File S2).

In Figure 5,the protein monomer surface of revolution is shown.

The spike protein monomer of SARS has a base surface with a radius of 8 units and a length of 1000 units on the *x*-axis. The envelope protein monomer of SARS-CoV-2 has a base surface radius of 125 units and a length of 1000 units. The membrane protein monomer of SARS-CoV-2 has a base radius of 22 units and a length of 1000 units. For the spike protein (SARS-CoV-2), the base of the surface has a radius of 12 units and a length of 1000 units on the x-axis. The termination of the surface of revolution after 1000 units of length is arbitrary, while its length is virtually infinite.

**Figure 6:**
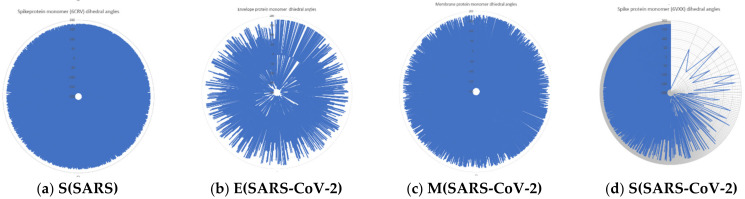
Radar plots of proteins monomers dihedral angles population (see also Supplementary Materials File S3).

Figure 6 shows radar plots of the dihedral angle populations for protein monomers.

The spike protein monomer of SARS has a uniform distribution of dihedral angles with upper values of 1800. The envelope protein of SARS-CoV-2 has some spatial regions uncovered by the dihedral angles. The membrane protein of SARS-CoV-2 has a partially uniform dihedral angle population which covers much of the conformational space. The spike protein of SARS-CoV-2 has a vast empty region where dihedral angle conformational space is not covered.

Polynomial trendline equations of Ramachandran plots obtained for the spike protein monomer of SARS and SARS-CoV-2, taking into account the energetically allowed regions, are represented in Table 4 (see also Supplementary Materials File S4).

**Figure 7:**
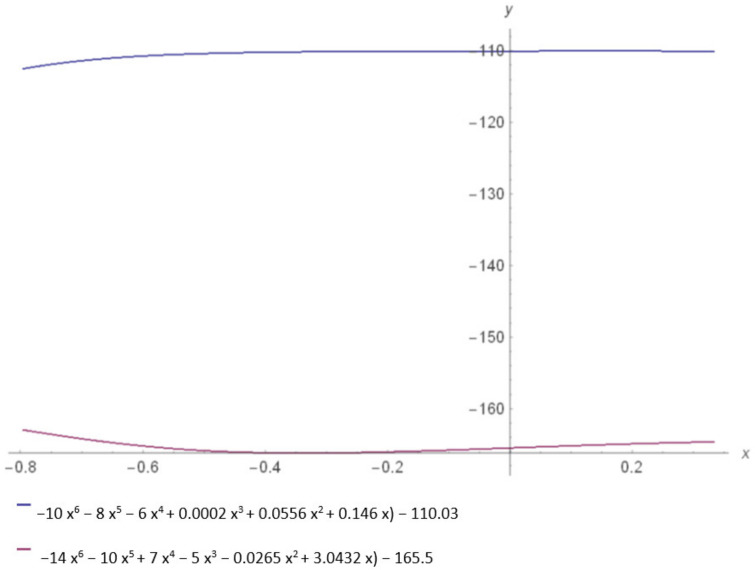
Spike protein energetically allowed regions represented by blue spike protein SARS and pink spike proteins SARS-CoV-2.

The complex roots were completed and turned to have a similar disposition, with six similar points disposed of from −1.5 to 1.5 in all four quadrants (Figure 7).

## 3. Discussion

Spike protein S is a high-priority target molecule in developing a vaccine or treatment against SARS-CoV-2. Many studies describe the interactions of spike protein S with some organic molecules. Some of them are driven by an experiment where a set of molecules are selected, and any interaction with the S protein is objectified. Others are computational studies. The benefit of most computational studies published is that they can probe a vast majority of the chemical space using, for example, virtual screening techniques. These virtual screening studies explore a multitude of binding sites. As stated before, a distinct and vast library of compounds is used. As expected, these results regarding energy (binding energy, Gibbs energy) are reported. This kind of computation does not offer a clear view of the phenomena. Usually, these results are expressed in kcal/mo. Sometimes, the only way to assess a compound’s attractiveness in terms of spike protein interaction is by searching for the lowest possible interaction energy. This kind of judgement offers a one-dimensional view of the process.

Also, computational studies regarding epitope–paratope interactions are to be noted. These studies usually used peptide libraries, and the interactions are treated in a ligand–receptor paradigm. Furthermore, structural correlations are hard to perform even using a fragment-based approach.

This computational study uses a multimodal representation of spike protein interactions with some fragment proteins. The interactions are represented using heat maps and radar plots. Also, the chemical space of each potential target (S, M, N, E SARS-CoV-2) protein is represented compared with the SARS-CoV-2 spike protein.

**Figure 8:**
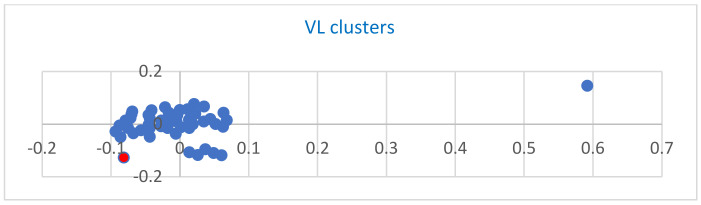
VL cluster. Two major groups of clusters are observed: a major group composed of VL: 2, 3, 4, 5, 6, 8, 10, 11, 12, 13, 14, 15, 16, 17, 18, 19, 20, 21, 22, 25, 26, 27, 28, 29, 30, 31, 32, 33, 34, 36, 37, 38, 39, 40, 41, 41, 43, 44, 46, 47; a small one composed of VL 7, 9, 23, 25, 45 and two single clusters 1 and 35.

The set of VL structures used to describe interactions with SARS-CoV-2 surface proteins is distinct. As seen in Figure 8, two significant groups of clusters are observed.

**Figure 9:**
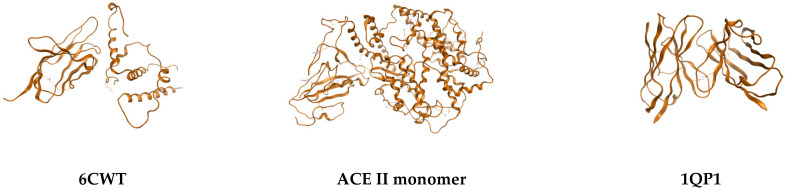
Most favorable interaction of fragment 47 (6CWT), represented with ACE II monomer and anther VL fragment 26with a polynomial discriminant value of 347,490 to demonstrate the specificity of VL—spike protein interaction and the lack of mass effect in this computational study.

Taking into account the polynomial discriminant as a unidimensional way of quantifying and judging spike protein S interactions with different molecular targets, it is observed that the value of VL (Aa polynomial discriminant (8099.72)) is close to the value of fragment 47 discriminant (41,645.3). The difference between the values of magnitude is 5.141. Theoretically, the Aa sequence 1 with the spike S protein is five times weaker than the interaction of fragment 47 (ACE II receptor, its ideal target) with the spike S protein. Virtually, viral capsules do not interact with each other. There are virtually no interactions between the molecules resulting from the final biosynthetic pathway. Furthermore, they have a particular way of rejecting each other. So, a minimum range of interaction in a monodimensional space, which has to be characterized by protein S interaction spaces, is set to be a spike protein S–spike protein S interaction as a lower limit (495.01) and a spike protein S–ACE II receptor interaction as an upper limit (41,645.3) (Figure 9).

On the function interval −0.5 ≤ x ≤ −0.15, function f1 = −14x^6^ − 10x^5^ + 7x^4^ − 5x^3^ − 0.0265x^2^ + 3.0432x − 165.5 is null, f1 = 0, and function f2 = 10x^6^ − 8x^5^ − 6x^4^ + 0.0002x^3^ + 0.0556x^2^ + 0.146x − 110.03 becomes f2 = −110, thus a straight line parallel to ox. The two straight lines on the interval (−0.5; −0.15) are parallel and symmetric from the axis y = −147.5. The area enclosed between the two functions, on the interval (−0.5, −0.15), has a rectangular shape. S = [−0.5 − (−0.15)] × [−165 − (−130)] = (−0.35) × (−35) = 1225. The convergence of the two functions is determined via the following relationship:

$\int_{a}^{\infty }f(x)dx=\underset{x\to \infty }{\lim}\int_{a}^{\infty }f(x)$ then f1(x)/(−x^6^) and (f1(x))/(−x^6^) is computed: (f1(x))/〖−x〗^6^ =10 + 8/x + 6/x^2^ − (0.0002)/x^3^ − (0.0556)/x^4^ − (0.146)/x^5^ + (110.3)/x^6^ also (f2(x))/(−x^6^) = 14 + 10/x − 7/x^2^ +5/x^3^ + (0.0265)/x^4^ − (3.0432)/x^5^ + (165.5)/x^6^. Because $\underset{x\to \infty }{\lim}{\frac{A}{x^{n}}=0}^{}$, where *A* is a constant, the limits of the two functions are 10 and 14, respectively. While the limits have positive values (10 > 0, 14 > 0), both of the functions have the following convergence domain: −∞ < x < +∞.

The Riemann theorem shows that via permutation of a series of terms, the sum can be made equal to any number given before. In the case of functions f1(x) and f2(x), these numbers are 10 and 14. So, the functions are convergent.

The surface of revolution in Euclidean space is a surface created by a rotating curve around an axis of rotation [44]. Here, the rotation axis originates at the object’s center in Euclidean space. Such surfaces generated by a straight line include cylindrical and conical surfaces. The surface of resolution coordinates expression is obtained by rotating a y = f(x) curve around the x-axis described by cylindrical coordinate r = f(z). In Cartesian coordinates, the parametrization in terms of z and θ is(f(z)cos(θ), f(z) sin (θ),z). If x and y are defined in terms of a parameter t, then a parametrization of t and θ is obtained. If x and y are functions (functions of proteins x,y,z atom coordinates) of t, then the surface of revolution obtained by revolving the curve around the x-axis is described in cylindrical coordinates by parametric equations (r,θ,z) = (y(t), θ, x(t)), and the surface of revolution obtained by revolving the curve around the *y*-axis is described by (r,θ,z)= (x(t), θ, y(t)). In Cartesian coordinates, these becomes (y(t)cos(θ), y(t)sin(θ), z(t) and (x(t)cos(θ), x(t)sin (θ), y(t)) [45,46]. Protein monomers have a distinct surface of revolution regarding the value, number, and topology of dihedral angles. It seems that the surface of revolution computed as a logarithmic equation dependent on molecule dihedral angles varies with the mass of the protein. Virtually, the SARS-CoV-2 surface proteins’ (spike, envelope, membrane) radius of revolution tends to converge in one point, in contrast with the spike protein of SARS, where the generators are parallel. Judging from the base of the surface of revolution, the envelope protein has the most extensive base (125 units).

**Figure 10:**
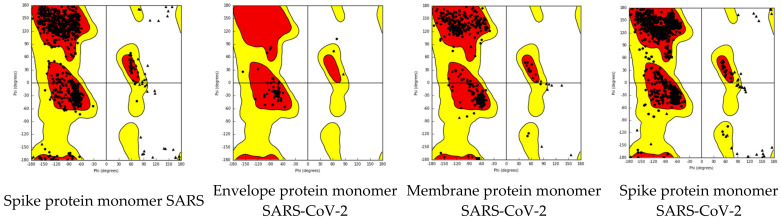
Ramachandran plots of viral proteins monomers. The spike protein of SARSA and SARS-CoV-2 have similar allowed regions. Antiparallel ß sheets, right-handed ἀ helix, and collagen triple helix are dominant in spike protein for both SARS and SARS-CoV-2.

**Figure 11:**
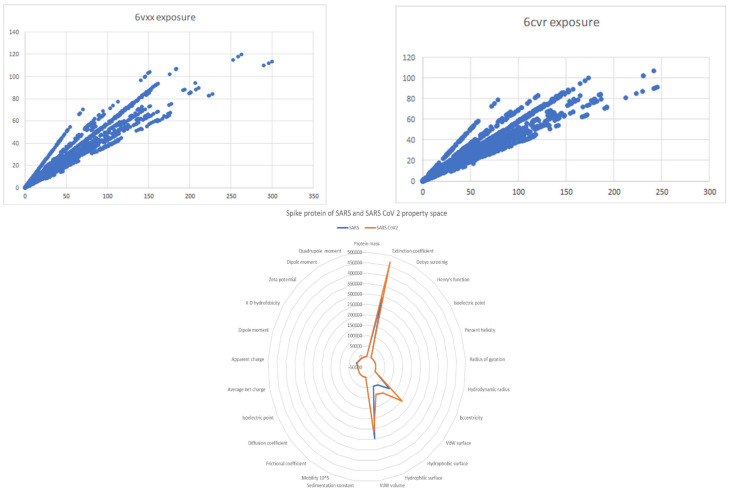
Property space of spike protein of SARS and SARS-CoV-2. Spike property space of SARS-CoV-2.

Ramachandran plots are a tool for visualizing energetically allowed domains for backbone dihedral angles ψ against ϕ of amino acid residues in proteins. Dihedral angle values are annular (0 degrees is the same as 360 degrees). Ramachandran plots warp from right to left and bottom to top [47]. The ω angle at the peptide bond is usually 180° since the partial-double-bond character keeps the peptide planar. The torsional angles of each fragment in a peptide define the geometry of its molecular connections to its adjacent residues by disposing of its planar peptide bond relative to the adjacent planar peptide bonds. The torsional angles characterize the conformation of the residues and the peptide. While somewhat energetically unfavorable, the specific geometry of functionally relevant residues may be essential for the protein’s function. Conformations need to be stabilized by the protein using H-bonds [48]. Figure 10 represents Ramachandran plots of the SARS and SARS-CoV-2 spike protein, envelope, and membrane protein of SARS-CoV-2. Spike protein monomer for SARS and SARS-CoV-2 seems to have similar energetically favorable domains rich in antiparallel beta-sheets, parallel beta-sheets, collagen triple helix, and right-handed alpha helix, thus emphasizing their similar spatial backbone constitution. Envelope and membrane protein monomers do not possess a rich structural complexity like the spike protein (Figure 11).

The property space of both spike proteins demonstrates similar characteristics of both molecules. Solvent exposure of both molecules shares common characteristics. Spike protein SARS and SARS-CoV-2 solvent exposure are similar (Figure 11).

Binding site identification of potential therapeutic targets is crucial in every drug design process as the expected spike protein has various binding sites. Also, many binding sites in various spike protein regions are described in the literature. An efficient vaccine can be designed by identifying the energetically and structurally favorable binding site. In the present study, the ligand’s interaction with its receptor (spike protein) is a general process of applying various binding sites.

Furthermore, the mirror symmetry of SARS-CoV-2 compared with SARS can potentially suggest potential ligands that can be used as vaccines. Symmetric molecules of SARS-proven ligands can be considered in such a way. Also, as seen in Figure 7, the difficulty of finding a proper ligand (organic molecule or Aa sequence) is due to the depleted dihedral angle region of SARS-CoV-2comparedwith SARS. Moreover, the symmetry of the two spike proteins suggests that reverse engineering (and thus retrosynthesis) is possible.

## 4. Materials and Methods

The design of an appropriate paratope that can lead to a COVID-19 antibody in in silico methods was used together with secondary data derived from the literature. In order to find a suitable paratope, a set of very low (VL) random sequences were used to assess the chemical space of epitope–paratope interactions (see also Supplementary Materials File S1).

Firstly, a target for a potential antibody was established. Spike protein S was chosen as a target due to its volume, shape, and frequency at the external membrane of COVID-19 compared to proteins E, M, and N, respectively. Computational studies and published data show that spike S protein is a valid target [49,50,51,52].

Also, an Aa sequence was chosen as an epitope. The Aa sequence was chosen from the literature (Nandy et al.) [53,54].

A set of 47 PDB VL chains was chosen from the literature to explore the paratope space by evaluating the epitope–paratope interactions using distinct computational techniques.

Firstly, a light chain of 47PDB structures was used. Paratopes were detected for each structure using the Paratome online engine [55]. Results were selected manually for Aa sequences to be at the outer portion of the light chain. Small and long Aa sequences were selected. After obtaining the sequence, they were computed using the Vendruscolo Lab software package named Parapred (https://www-cohsoftware.ch.cam.ac.uk, accessed on 1 July 2023). For example, the following sequence was obtained for 1A8J using Paratome: SYEGSDF. After checking that the sequence was of the outer external region and could contact the antigen, the probabilistic contribution of each Aa was computed using Parapred, in this case yielding the following values: S 0.1649, Y 0.486472, E 0.713605, G 0.32135, S 0.469778, and F 0.0840889. In order to take into account the epitopes’ first light chains, interactions with the light chains were studied. The PSOPIA online server was used [56]. PSOPIA computes three types of scores: sequences, similarities, domain–domain interactions, Aa contacts (sum of edge weights, the shortest path between homologous proteins in a protein–protein interaction network), and a score which is the sum of all three scores, respectively. After computing, the interaction Paratome server was used for selecting the paratopes [51].

iFrag, a protein–protein interaction prediction server, was used to compute and quantify the interaction between epitopes and paratopes [52]. First, the row sequences of the epitopes and paratopes were introduced. Below are the spike S protein monomer and ACEE II monomer sequences.

Spike protein monomer:

AYTNSFTRGVYYPDKVFRSSVLHSTQDLFLPFFSNVTWFHAIHDNPVLPFNDGVYFASTE

KSNIIRGWIFGTTLDSKTQSLLIVNNATNVVIKVCEFQFCNDPFLGVNCTFEYVSFKNLR

EFVFKNIDGYFKIYSKHTPINLVRDLPQGFSALEPLVDLPIGINITRFQTLLALHAAYYV

GYLQPRTFLLKYNENGTITDAVDCALDPLSETKCTLKSFTVEKGIYQTSNFRVQPTESIV

RFPNITNLCPFGEVFNATRFASVYAWNRKRISNCVADYSVLYNSASFSTFKCYGVSPTKL

NDLCFTNVYADSFVIRGDEVRQIAPGQTGKIADYNYKLPDDFTGCVIAWNSNNLDSKGNY

NYLYRKPFERDIYFPLQSYGFQPTNVGYQPYRVVVLSFELLHAPATVCGPKKSTNLVKNK

CVNFNFNGLTGTGVLTESNKKFLPFQQFGRDIADTTDAVRDPQTLEILDITPCSFGGVSV

ITPGTNTSNQVAVLYQDVNCTEVNVFQTRAGCLIGAEHVNNSYECDIPIGAGICASYQTS

QSIIAYTMSLGAENSVAYSNNSIAIPTNFTISVTTEILPVSMTKTSVDCTMYICGDSTEC

SNLLLQYGSFCTQLNRALTGIAVEQDKNTQEVFAQVKQIYKTPPIKDFGGFNFSQILPDP

SKPSKRSFIEDLLFNKVTKFNGLTVLPPLLTDEMIAQYTSALLAGTITSGWTFGAGAALQ

IPFAMQMAYRFNGIGVTQNVLYENQKLIANQFNSAIGKIQDSLSSTASALGKLQDVVNQN

AQALNTLVKQLSSNFGAISSVLNDILSRLDPPEAEVQIDRLITGRLQSLQTYVTQQLIRA

AEIRASANLAATKMSECVLGQSKRVDFCGKGYHLMSFPQSAPHGVVFLHVTYVPAQEKNF

TTAPAICHDGKAHFPREGVFVSNGTHWFVTQRNFYEPQIITTDNTFVSGNCDVVIGIVNN

TVYDPLQPELDS

ACEII monomer

STTEELAKTFLETFNYEAQELSYQSSVASWNYNTNITEENVQNMNNAGDKWSAFLKEQST

LAQTYPLQEIQNLTVKLQLQALQQNGSSVLSEDKSKRLNTILNTMSTIYSTGKVCNPDNP

QECLLLEPGLNEIMANSLDYNERLWAWESWRSEVGKQLRPLYEEYVVLKNEMARANHYED

YGDYWRGDYEVNGVDGYDYSRGQLIEDVEHTFEEIKPLYEHLHAYVRAKLMNAYPSYISP

IGCLPAHLLGDMWGRFWTNLYSLTVPFGQKPNIDVTDAMVDQAWDAQRIFKEAEKFFVSV

GLPNMTQGFWENSMLTDPGNVQKAVCHPTAWDLGKGDFRILMCTKVTMDDFLTAHHEGH

IQYDMAYAAQPFLLRNGANEGFHEAVGEIMSLSAATPKHLKSIGLLSPDFQEDNETEINF

LLKQALTIVGTLPFTYMLEKWRWMVFKGEIPKDQWMKKWWEMKREIVGVVEPVPHDETYC

DPASLFHVSNDYSFIRYYTRTLYQFQFQEALCQAAKHEGPLHKCDISNSTEAGQKLFNML

RLGKSEPWTLALENVVGAKNMNVRPLLNYFEPLFTWLKDQNKNSFVGWSTDWSPYADPFG

EVFNATKFPSVYAWERKKISNCVADYSVLYNSTFFSTFKCYGVSATKLNVYADSFVVKGD

DVRQIAPGQTGVIADYNYKLPDDFMGCVLAWNTRNIDATSTGNYNYKYRYLRHGKLRPFE

RDISNVPFSPDGKPCTPPALNCYWPLNDYGFYTTTGIGYQPYRVVVLSFE

In order to characterize the VL sequences, a cluster analysis was performed using molecular descriptors. The properties, after the cluster was computed, are the following: sum of atomic polarizabilities, number of hydrogen bond acceptors, number of acidic atoms, number of aromatic atoms, number of H donor atoms, the sum of the number of H bond acceptors and donors, number of heavy atoms, information content, medium information content, number of carbon atoms, number of hydrogen atoms, number of nitrogen atoms, number of oxygen atoms, Balaban index, difference in bonded atom polarizabilities, number of rotable single bonds, number of aromatic bonds, number of bounds, number of heavy bounds, number of single bounds, atomic connectivity index, atomic valence connectivity index, number of chiral centes, number of unconstrained chiral centers, density, diameter (most considerable vertex eccentricity in a graph), SlogP, molar refractivity, topological polar surface area, vertex adjency information, volume surface area, van der Waals acceptor surface area, molecular weight, winner path, Weiner polarizability, Zagreb index, surface rugosity, total energy, angular energy, electronic energy, non-bonded energy, solvation energy, strain energy, van der Waals energy, and globularity. As seen in Figure 8, VL fragment 1 (6CVR, colored in red) is located outside the cluster together with an electric VL fragment 35. Fragments 46 and 47 are located inside the significant (central) cluster.

The following PDB models were used: 6VXXfor SARS-CoV-2spike protein, 6CVR for SARS spike protein, 5X29for SARS-CoV-2 envelope protein, and3I6Gfor SARS-CoV-2 spike protein. Furthermore, the PDB models were energetically minimized, charges corrected, and protonated at pH 7.4, a salt concentration of 0.9 nmol/L, and at 315 K. The model preparations were performed using Schrodinger2009 and MOE 2009software packages [57,58]. Shape-derived descriptors were computed for all the PDB structures and computed to define protein shape and functionality consecutively. The lowest energy conformations were retained for each protein. The following descriptors were used:pmi(y), Kier1,2,3, KierA1,2,3, first, second, and third alpha-modified shape index, normalized PMI ratio. The descriptors were computed using the MOE 2009 software package.

In order to reduce the amount of data and to simplify the results, monomers for each protein(envelope protein, membrane protein, spike protein) were prepared computationally, as stated before. The trimers and pentamer PDB structures were energetically minimized, and charges were corrected using the AMBER force field available in MOE 2009 software package. For each monomer structure, the energy was minimized, charges corrected, and structures protonated at a physiological pH. Dihedral (torsion angles) for each monomer were computed using the Chemoffice 2008 software package.

In probing the chemical space, some structural information, from simple dihedral angles to complex tertiary and quaternary organizations, was obtained computationally using Schrodinger 2009 software packages. Dihedral angles were represented using a scatter plot for each monomer (oy dihedral angle values; ox atom pairs composing the planes for dihedral angle). While an abrupt cut-off was observed in data, and an abrupt variation in data was noted, a logarithmic scale was used. Also, as discussed in the introductory part, by normalizing the data, clusters are more easily observed (Figure 2). A logarithmic trendline was further calculated using the Microsoft Office 2019 software package for the discussed monomers.

In order to expand data dimensionality, a Ramachandran plot diagram was computed for all four monomers in order to characterize the dihedral angles of the structures using the Schrodinger 2009 software package. The Wolfram Alpha software (https://www.wolframalpha.com/, accessed on 1 July 2023) interface was used to perform a cluster analysis of all dihedral angles. To compare their function domains, the function table d^n^/dx^n^ (f(x)) for n = 1 … 4 was calculated for all four monomers. Surface plots were computed using the radius of gyration of each logarithmic trend line. Here, r2, while exploring a trend and not a correlation between two phenomena, is not significant. A histogram was computed using dihedral angle values on 300 intervals for all three monomers. Using logarithmic trendline equations, a derivate equation was computed for all three protein monomers to represent the dynamic of dihedral angles as a function graphically.

Radar plots were used to emphasize the dihedral angles of protein monomers. Furthermore, the surface of revolution for protein S for SARS and for M, E, and S for SARS-COV-2 were computed to retrieve the 3D dimensionality of monomers’ dihedral angles. Surfaces were generated using the following formulas: for S (SARS) protein monomer table: d^n^/dx^n^ (−0.059 log(x) + 6.1779) for n = 1 … 4; for M (COVID-19) protein monomer table: d^n^/dx^n^(−1.51011 log(x) + 6.1779) for n = 1 … 4; for E (COVID-19), protein monomer table: d^n^/dx^n^(−0.842 log(x) + 62.544) for n = 1 … 4; and for S (COVID-19) protein monomer table: d^n^/dx^n^(−0.443 log(x) + 9.7581) for n = 1 … 4.

## 5. Conclusions

The dimensionality of the COVID-19 interaction space is defined by its interaction with its core receptor. The interaction space has a finite dimensionality defined quantitatively by the polynomial discriminant. Judging by its 2D dimensionality, the interaction space has a negative and positive domain through which epitope–paratope interactions are defined.

SARS and SARS-CoV-2 spike proteins have similar solvent exposure and mirror symmetry regarding their dihedral angle chemical space. Both proteins have a similar solvent exposure even if their dihedral angle conformational space has mirror symmetry. Mirror symmetry explains to some extent the chemical inaccessibility of the spike protein SARS-CoV-2 as a receptor and the evasive but permanent chemical bonding in which it is involved.

## Figures and Tables

**Table 1:** Structures and interaction equations.

Nr	Structure	Polynomial Eq.	Logarithmic Eq.	Polynomial Discriminant Eq. Solution	Logarithmic Eq. Solution
1	6cvr	y = −21x^6^ − 16x^5^ − 12x^4^ − 0.9x^3^ − 0.5x^2^ + 0.0226x − 2.6132	y = −0.964 ln(x) + 8.6756	−1.25027 × 10^13^	8099.72
2	5M76	y = −19x^6^ − 15x^5^ − 12x^4^ − 0.8x^3^ − 0.6x^2^ − 0.0007x + 0.0118	y = 0.2959 ln(x) − 1.43	1.75267 × 10^9^	125.551
	5M6A	y = −19x^6^ − 15x^5^ − 11x^4^ − 0.8x^3^ − 0.5x^2^ − 0.001x + 0.0703	y = 0.1133 ln(x) − 0.0932	3.13803 × 10^9^	2.2764
4	5C9K	y =−19x^6^ − 15x^5^ − 12x^4^ − 0.9x^3^ − 0.6x^2^ + 0.0028x − 0.1209	y = 0.1457 ln(x) − 0.2645	−1.74887 × 10^10^	6.14337
5	5ACM	y = −19x^6^ − 16x^5^ − 12x^4^ − 0.9x^3^ − 0.6x^2^ + 0.0023x − 0.0865	y = 0.1653 ln(x) − 0.7071	−1.20161 × 10^10^	72.0728
6	4X4Z	y = −19x^6^ − 15x^5^ − 11x^4^ − 0.8x^3^ − 0.6x^2^ + 0.0021x + 0.4072	y = −0.039 ln(x) + 1.4726	7.63376 × 10^10^	25,033,075,340,170,460
7	4N1C	y = −19x^6^ − 15x^5^ − 12x^4^ − 0.9x^3^ − 0.7x^2^ + 0.0015x + 0.0317	y = −0.022 ln(x) + 0.4792	1.26141 × 10^10^	2.8822 × 10^9^
8	4LVE	y = −19x^6^ − 15x^5^ − 12x^4^ − 0.9x^3^ − 0.6x^2^ − 0.0017x + 0.2568	y = 0.1423 ln(x) − 0.33	3.61698 × 10^10^	10.166
9	4K07	y = −20x^6^ − 16x^5^ − 13x^4^ − 1.0x^3^ − 0.6x^2^ + 0.0028x + 0.4214	y = −0.105 ln(x) + 1.5204	5.92218 × 10^10^	1.9435 × 10^6^
10	4K3G	y = −19×^6^ − 15x^5^ − 11x^4^ − 0.8x^3^ − 0.5x^2^ − 0.0022x + 0.1699	y = 0.2815 ln(x) − 1.1125	7.39881 × 10^9^	52.0416
11	4BJL	y = −19x^6^ − 15x^5^ − 12x^4^ − 0.8x^3^ − 0.5x^2^ − 0.0021x + 0.1734	y = 0.0611 ln(x) − 0.0185	7.84012 × 10^9^	1.35362
12	3UPA	y = −19x^6^ − 15x^5^ − 12x^4^ − 0.9x^3^ − 0.6x^2^ + 0.0047x + 0.3328	y = −0.067 ln(x) + 1.6866	4.86836 × 10^10^	8.56157 × 10^10^
13	3T0W	y = −19x^6^ − 15x^5^ − 11x^4^ − 0.8x^3^ − 0.6x^2^ + 0.001x − 0.009	y = 0.1872 ln(x) − 0.4948	−1.39331 × 10^9^	14.0576
14	3MCG	y = −20x^6^ − 17x^5^ − 12x^4^ − 0.9x^3^ − 0.6x^2^ + 0.0018x − 0.0765	y = 0.1462 ln(x) − 0.641	−1.241 × 10^10^	80.1905
15	3CDC	y = −20x^6^ − 16x^5^ − 13x^4^ − 0.9x^3^ − 0.6x^2^ + 0.0037x + 0.4392	y = −0.119 ln(x) + 1.6773	8.86941 × 10^10^	1.3224 × 10^6^
16	3BJL	y = −19x^6^ − 15x^5^ − 11x^4^ − 0.8x^3^ − 0.5x^2^ − 0.0045x + 0.3602	y = 0.1276 ln(x) − 0.0371	1.71228 × 10^10^	1.33743
17	3BDX	y = −19x^6^ − 15x^5^ − 11x^4^ − 0.8x^3^ − 0.5x^2^ − 0.0016x + 0.1355	y = 0.3303 ln(x) − 1.4623	5.93645 × 10^9^	83.6957
18	3BD3	y = −19x^6^ − 15x^5^ − 11x^4^ − 0.8x^3^ − 0.5x^2^ + 0.0059x − 0.1529	y = 0.2444 ln(x) − 0.7981	−7.37006 × 10^9^	26.1945
19	3B5G	y = −19x^6^ − 15x^5^ − 11x^4^ − 0.8x^3^ − 0.6x^2^ − 0.0014x + 0.1304	y = 0.3172 ln(x) − 1.4154	2.09459 × 10^10^	86.6753
20	2Q2O	y = −20x^6^ − 16x^5^ − 12x^4^ − 0.9x^3^ − 0.5x^2^ + 0.0062x + 0.3398	y = −0.093 ln(x) + 1.6544	1.34625 × 10^10^	5.31829 × 10^7^
21	2OMB	y = −19x^6^ − 15x^5^ − 12x^4^ − 0.8x^3^ − 0.6x^2^ − 0.5x + 0.0285	y = 0.1881 ln(x) − 0.6922	2.85107 × 10^12^	39.6447
22	2MCG	y = −20x^6^ − 17x^5^ − 12x^4^ − 0.9x^3^ − 0.6x^2^ + 0.0018x − 0.0765	y = 0.1462 ln(x) − 0.641	−1.241 × 10^10^	80.1905
23	2KQN	y = − 19x^6^ − 15x^5^ − 12x^4^ − 0.8x^3^ − 0.5x^2^ + 0.0063x + 0.2908	y = −0.131 ln(x) + 1.8406	1.4796 × 10^10^	1.26475 × 10^6^
24	2KQM	y = − 20x^6^ − 16x^5^ − 13x^4^ − 1.1x^3^ − 0.6x^2^ + 0.001x + 0.1356	y = −0.034 ln(x) + 0.4816	1.60522 × 10^10^	1.41793 × 10^6^
25	1REI	y = −19x^6^ − 15x^5^ − 12x^4^ − 0.9x^3^ − 0.6x^2^ + 0.0047x + 0.0759	y = −0.099 ln(x) + 1.7129	1.07351 × 10^10^	3.26717 × 10^7^
26	1QP1	y = −20x^6^ − 16x^5^ − 12x^4^ − 0.9x^3^ − 0.6x^2^ + 0.0013x + 0.161	y = −0.053 ln(x) + 0.6762	2.62376 × 10^10^	347,490
27	1QAC	y = −20x^6^ − 16x^5^ − 12x^4^ − 0.9x^3^ − 0.6x^2^ − 0.0003x + 0.0601	y = 0.0241 ln(x) + 0.0217	9.87188 × 10^9^	0.406401
28	1MCW	y = −20x^6^ − 16x^5^ − 12x^4^ − 0.9x^3^ − 0.6x^2^ + 0.002x − 0.0665	y = −0.006 ln(x) + 0.2463	−1.11613 × 10^10^	672,640,970,952,404,352
29	1MCS	y = −19x^6^ − 16x^5^ − 13x^4^ − 0.9x^3^ − 0.6x^2^ + 0.0014x − 0.0533	y = 0.1255 ln(x) − 0.5198	−6.94971 × 10^9^	62.918
30	1MCJ	y = −19x^6^ − 16x^5^ − 12x^4^ − 1.0x^3^ − 0.6x^2^ + 0.0012x − 0.0402	y = 0.1337 ln(x) − 0.5691	−5.368 × 10^9^	70.5657
31	1MCI	y = −19x^6^ − 16x^5^ − 13x^4^ − 1.0x^3^ − 0.6x^2^ + 0.0013x − 0.0482	y = 0.1293 ln(x) − 0.5426	−6.26192 × 10^9^	66.4495
32	1MCD	y = −19x^6^ − 16x^5^ − 12x^4^ − 1.0x^3^ − 0.6x^2^ + 0.0012x − 0.0402	y = 0.1337 ln(x) − 0.5691	−5.368 × 10^9^	70.5657
33	1MCC	y = −19x^6^ − 16x^5^ − 13x^4^ − 0.9x^3^ − 0.6x^2^ + 0.0014x − 0.0533	y = 0.1255 ln(x) − 0.5198	−6.94971 × 10^9^	62.918
34	1MAJ	y = −19x^6^ − 15x^5^ − 12x^4^ − 0.9x^3^ − 0.6x^2^ − 0.0005x + 0.126	y = 0.0665 ln(x) − 0.1725	1.75923 × 10^10^	13.383
35	1LGV	y = −19x^6^ − 15x^5^ − 11x^4^ − 0.8x^3^ − 0.5x^2^ − 0.0018x + 0.0735	y = 0.1481 ln(x) − 0.6409	3.27612 × 10^9^	75.7533
36	1IVL	y = −20x^6^ − 16x^5^ − 12x^4^ − 0.9x^3^ − 0.7x^2^ + 0.0015x + 0.092	y = 0.0212 ln(x) + 0.333	4.36258 × 10^10^	1.50764 × 10^−7^
37	1EK3	y = −19x^6^ − 15x^5^ − 12x^4^ − 0.9x^3^ − 0.6x^2^ + 0.0002x + 0.1087	y = 0.0677 ln(x) − 0.0314	1.52104 × 10^10^	1.59012
38	1EEU	y = −19x^6^ − 15x^5^ − 12x^4^ − 0.8x^3^ − 0.6x^2^ − 0.0013x + 0.1335	y = 0.0796 ln(x) − 0.1689	2.13078 × 10^10^	8.34664
39	1EEQ	y = −19x^6^ − 15x^5^ − 12x^4^ − 0.8x^3^ − 0.6x^2^ − 0.0013x + 0.1344	y = 0.098 ln(x) − 0.2921	2.14642 × 10^10^	19.6999
40	1DCL	y = −20x^6^ − 16x^5^ − 13x^4^ − 0.9x^3^ − 0.6x^2^ + 0.002x − 0.0963	y = 0.1157 ln(x) − 0.4593	−1.51605 × 10^10^	52.9713
41	1BWW	y = −20x^6^ − 16x^5^ − 13x^4^ − 1.0x^3^ − 0.6x^2^ + 0.0013x + 0.0516	y = −0.03 ln(x) + 0.4975	7.65811 × 10^9^	1.59239 × 10^7^
42	1BJM	y = −18x^6^ − 14x^5^ − 10x^4^ − 0.7x^3^ + 0.0002x^2^ − 0.0464x + 2.9016	y = −0.03 ln(x) + 1.5043	1.58331 × 10^13^	59,837,435,299 × 10^−11^
43	1B6D	y = −19x^6^ − 15x^5^ − 12x^4^ − 0.9x^3^ − 0.7x^2^ + 0.0014x + 0.1079	y = −0.057 ln(x) + 0.7972	4.37707 × 10^10^	1.18584 × 10^6^
44	1BOW	y = −21x^6^ − 17x^5^ − 13x^4^ − 1.0x^3^ − 0.6x^2^ + 0.0016x + 0.2476	y = −0.074 ln(x) + 1.0069	3.80249 × 10^10^	811.595
45	1A8J	y = 19x^6^ − 16x^5^ − 12x^4^ − 0.9x^3^ − 0.6x^2^ + 0.0023x − 0.0865	y = 0.1653 ln(x) − 0.7071	1.41204 × 10^8^	72.0728
46	6VXX	y = −19x^6^ − 15x^5^ − 11x^4^ − 0.7x^3^ − 0.0002x^2^ + 0.119x − 10.705	y = −2.722 ln(x) + 23.157	−1.72453 × 10^16^	4951.01
47	ACEII 6VXX	y = −16x^6^ − 12x^5^ − 9x^4^ − 0.6x^3^ − 0.0001x^2^ + 0.1x − 9.857	y = 0.0628 ln(x)−0.668	−4.79692 × 10^15^	41,645.3
48	6VXX 6VXX	y = −27x^6^ − 20x^5^ − 15x^4^ − 2.4x^3^ − 0.0001x^2^ +0.1x − 17.1143	y = 0.0053 ln(x) − 0.0593	−6.91299 × 10^17^	723,07.2

**Table 2:** Paratope sequences retrieved computationally.

ABR L1: QSIGNALA (27–34)
ABR L2: LLISAGSNLAS (46–56)
ABR L3: LGTYSAIDRA (89–98)

**Table 3:** Logarithmic trendline equations for each protein monomer dihedral angle.

Protein Monomer	Equations
S(SARS)	y = −0.059 ln(x) + 6.1779
E(SARS-CoV-2)	y = −8.42 ln(x) + 62.544
M(SARTS-CoV-2)	y = 1.5101 ln(x) − 8.9329
S (SARS-CoV-2)	y = −0.443 ln(x) + 9.7581

**Table 4:** Polynomial equations of energetically allowed regions of spike protein monomer.

Protein Monomer Energetically Allowed Regions	Equations
S (SARS)	y = −14x^6^ − 10x^5^ − 7x^4^ − 5x^3^ − 0.0265x^2^ + 3.0432x − 165.5
S (SARS-CoV-2)	y= −10x^6^ − 8x^5^ − 6x^4^ + 0.0002x^3^ + 0.0556x^2^ + 0.146x − 110.03

## Data Availability

On reasonable demand.

## Supplementary Materials

The supporting information can be downloaded at: https://www.mdpi.com/article/10.3390/ijms241512058/s1.
