# Supplementary material for: SARS-CoV-2 Spike Protein Interaction Space
Source: Int J Mol Sci. 2023 Jul 27;24(15):12058. doi: 10.3390/ijms241512058 (PMC10418891; doi:10.3390/ijms241512058)
Supplement: Supplementary file 1 [file ijms-24-12058-s001.zip › Supplementary Materials File S3.pdf]

**Supplimental Material File S3**

**Protein monomers dihedral angles clusters**

| Protein | Histogram of protein monomers dihedral angles | |
|---|---|---|
| **S** | 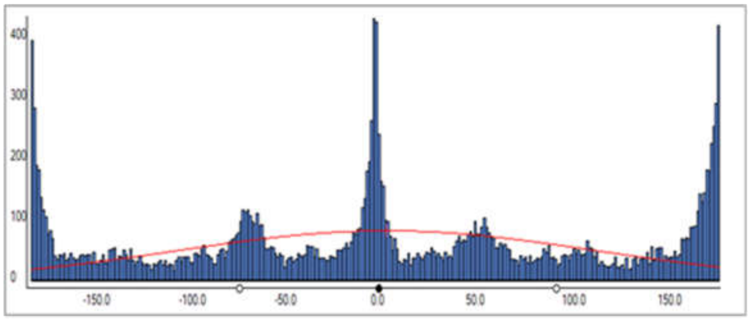 | |
| **E** | 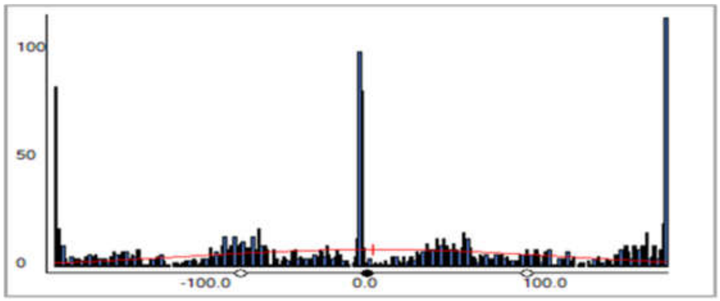 | |
| **M** | 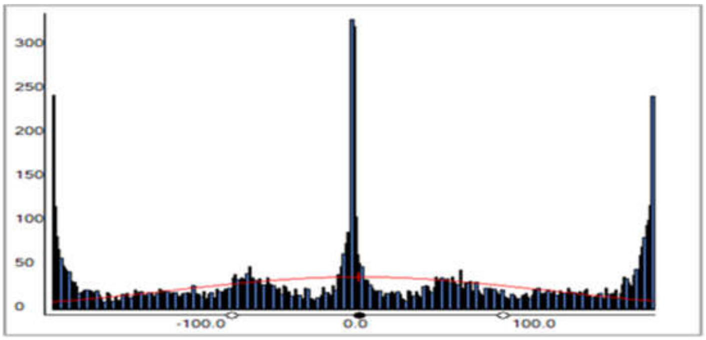 | |
| **6vxx** | 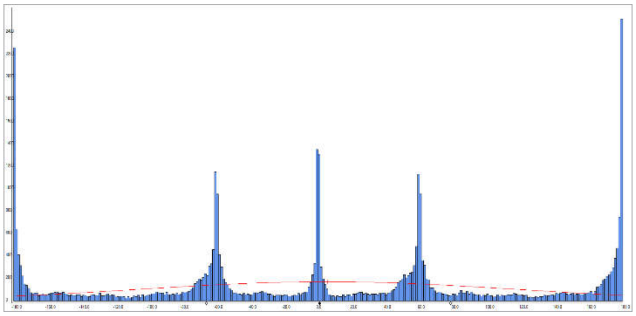 | |
