# Supplementary material for: SARS-CoV-2 Spike Protein Interaction Space
Source: Int J Mol Sci. 2023 Jul 27;24(15):12058. doi: 10.3390/ijms241512058 (PMC10418891; doi:10.3390/ijms241512058)
Supplement: Supplementary file 1 [file ijms-24-12058-s001.zip › Supplementary Materials File S4.pdf]

Supplementary Materials File S4

Complex roots of energetically allowed regions equations

Spike protein monomer S SARS

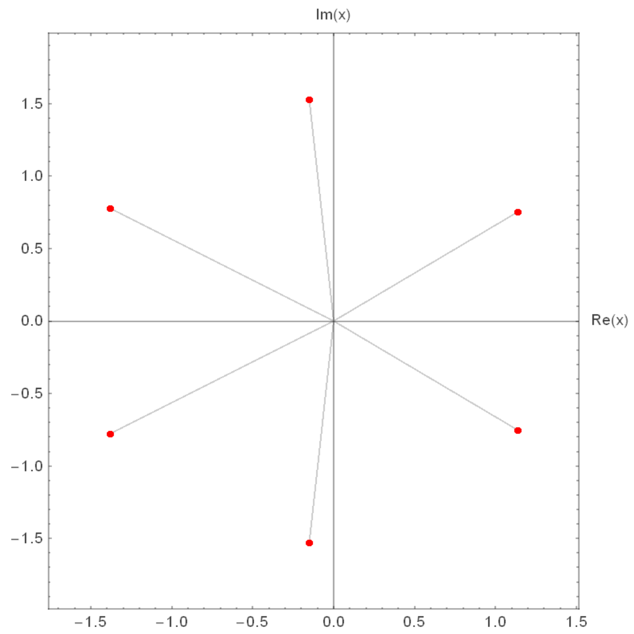

Spike protein monomer S SARS-CoV-2

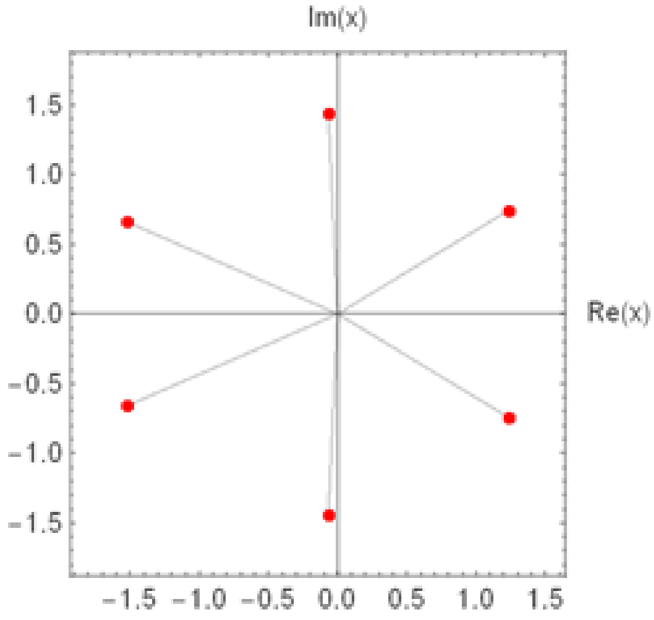

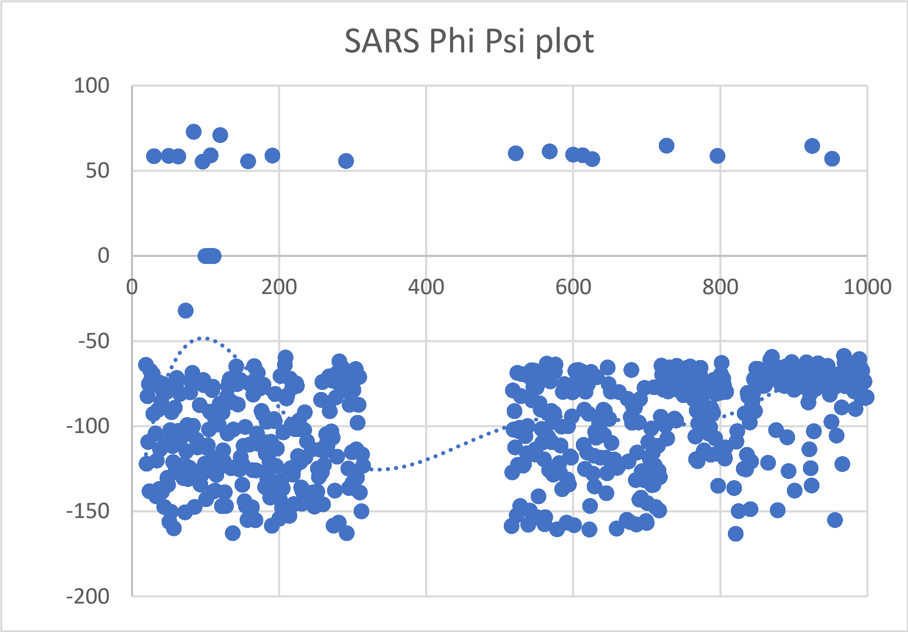
SARS Phi Psi plot
100
50
0
-50
-100
-150
-200
0
200
400
600
800
1000

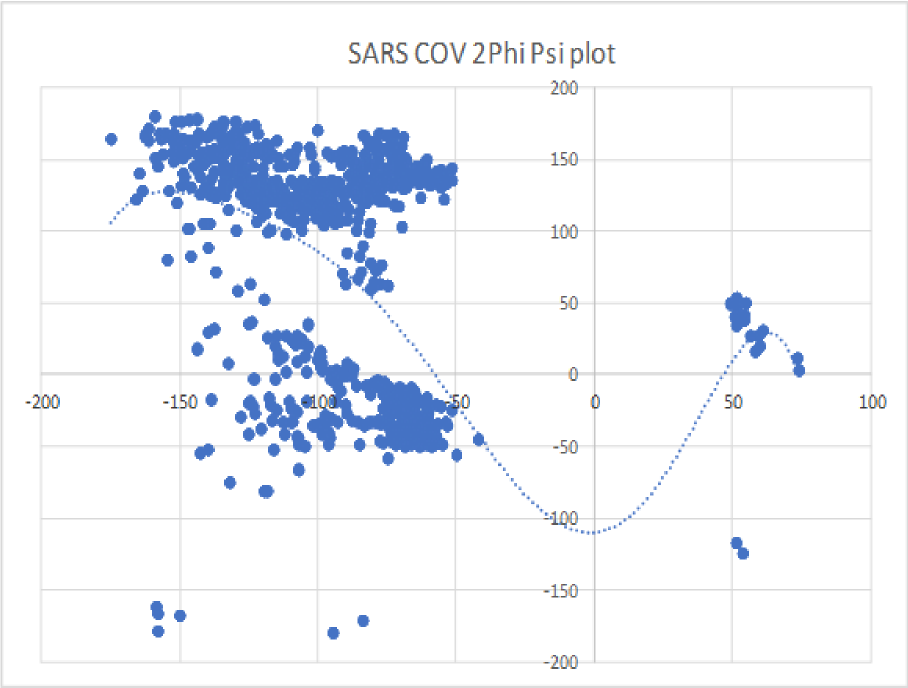
SARS COV 2 Phi Psi plot
200
150
100
50
0
-50
-100
-150
-200
-200
-150
-100
-50
0
50
100
